# Patient perceptions regarding ambulatory knee arthroplasties in China

**DOI:** 10.1186/s42836-025-00316-z

**Published:** 2025-06-09

**Authors:** Guanghui Zhao, Chengyuan Ma, Jianbing Ma, Jianpeng Wang

**Affiliations:** Department of Joint Surgery, Honghui Hospital, Xi’an Jiaotong University, No.555 East Youyi Road, Xi’an, 710054 Shaanxi China

**Keywords:** Perception, Ambulatory, Unicompartmental knee arthroplasty, Total knee arthroplasty

## Abstract

**Background:**

While same-day discharge models for knee arthroplasty have gained significant traction in China’s evolving healthcare landscape, patient perspectives on ambulatory surgical pathways remain underexplored. This qualitative study addresses a critical gap in the literature by systematically assessing patient experiences and perceptions regarding knee arthroplasty within China’s emerging ambulatory care framework.

**Methods:**

A prospective cohort of 195 consecutive patients scheduled for primary knee arthroplasty at a tertiary orthopedic referral center underwent structured data collection through the WenJuanXing platform between January 1 and June 1, 2024. This cross-sectional survey employed an anonymous voluntary survey instrument administered at two critical timepoints: 1) prior to any clinical discussions regarding postoperative care pathways, and 2) before initiation of standardized preoperative education protocols.

**Results:**

In total, 188 participants (96%, 188/195) completed the survey. Of them, 70% were female and 84% were 60 years or older. While 68% were familiar with ambulatory surgery, awareness did not significantly differ by age (*P* = 0.64), sex (*P* = 0.19), occupation (*P* = 0.42), location (*P* = 0.55), or education level (*P* = 0.81). Interestingly, only 8 patients anticipated discharge within 24 h post-surgery, with most (71.8%) expecting a 3-day or more stay. However, if postoperative care was assured, 66% expressed comfort with same-day or 24-h discharge. 93% considered ambulatory knee arthroplasty suitable, and 71.8% believed it would yield superior outcomes through quicker recovery and reduced complications, infections, and pain. Despite this optimism, only 45% were willing to endure longer waits, and a third were open to paying more or traveling farther for ambulatory knee arthroplasty.

**Conclusion:**

The study reveals that most Chinese patients initially want ≥ 3-day stays but may accept 24-h discharge for knee arthroplasty. One-third are unaware of ambulatory knee arthroplasty, so more education is needed as procedures rise.

**Supplementary Information:**

The online version contains supplementary material available at 10.1186/s42836-025-00316-z.

## Introduction

China’s joint arthroplasty surgery volume demonstrated sustained growth, reaching 951,986 procedures in 2019, with unicompartmental and total knee arthroplasties (UKA and TKA) constituting 40.56% of this surgical burden [1]. This epidemiological trajectory stems from three systemic drivers: (1) an expanding geriatric demographic requiring degenerative joint intervention; (2) persistent influx of incident osteoarthritis cases; and (3) critical shortages in both specialized arthroplasty centers and board-certified joint reconstruction surgeons [2, 3]. Compounding these pressures, the 2022 National Healthcare Security Administration prosthesis price negotiations achieved 82% average cost reductions for artificial joints, disproportionately increasing surgical accessibility among pensioners while exposing structural limitations in perioperative care capacity [4].

In recent years, the implementation of rapid recovery clinical pathways for joint arthroplasty has significantly reduced the length of hospital stays. These protocols have enabled the transition to ambulatory surgery, which has been proven to be safe and cost-effective for appropriately selected patients, thereby gaining increasing acceptance in the United States [5–11]. According to the China Ambulatory Surgery Alliance (CASA), ambulatory surgery is defined as “a surgical model wherein patients are admitted to the hospital, undergo surgery, and are discharged within one working day,” excluding outpatient surgeries performed in clinics or hospitals. In other words, surgeries that traditionally required multiple days of hospitalization can now be completed within 24 h through the ambulatory surgery approach [12]. As a member of the alliance and one of the leading joint arthroplasty centers in China, our institution initiated ambulatory joint arthroplasty in January 2023. Over the past year, more than 2,000 ambulatory surgeries have been successfully performed, achieving high levels of patient satisfaction.

While previous studies in the United States had explored patient and public perceptions of outpatient joint arthroplasty, revealing a preference for inpatient procedures among the majority of the public, with only a minority expecting same-day discharge, no research has yet investigated patient perspectives on ambulatory UKA or TKA in China [13, 14]. Prior to the absolute adoption of ambulatory surgery, it is important to better understand patients’ attitudes towards this practice. Increased knowledge of patients’ values allows for evidence-based, patient-centered perioperative care. This study aimed to gain insights into patient attitudes and views regarding UKA/TKA procedures conducted in an ambulatory setting. This study hypothesized that most of the patients would prefer ambulatory knee arthroplasties to inpatient, and most preferred to be discharged within 24 h after surgery.

While prior studies in the United States have examined patient and public perceptions of outpatient joint arthroplasty, indicating a preference for inpatient procedures among most of the population, with only a minority expecting same-day discharge, no research to date has explored patient perspectives on ambulatory UKA or TKA in China [13, 14]. Before fully embracing ambulatory surgery, it is crucial to gain a deeper understanding of patients'attitudes toward this approach. Enhanced knowledge of patients’ values can facilitate evidence-based, patient-centered perioperative care. The objective of this study was to investigate patient attitudes and views regarding UKA/TKA procedures performed in an ambulatory setting. This study hypothesized that most patients would prefer ambulatory knee arthroplasties over inpatient procedures and would favor discharge within 24 h post-surgery.

## Material and methods

Between January 1 st, 2024 and June 1 st, 2024, all patients scheduled for primary UKA or TKA at a tertiary orthopaedic hospital offering ambulatory knee arthroplasty services were invited to participate in a comprehensive 30-question survey administered via WenJuanXing (Ranxing Information Technology Co., Ltd., Changsha, China), a widely recognized online survey platform in China (see Appendix A). The inclusion criteria comprised: patients aged 18 years or older who were scheduled for primary UKA or TKA and willing to participate in the questionnaire survey. Patients unable to complete the questionnaire due to various reasons were excluded from the study. This investigation specifically aimed to elucidate patients’ perceptions of ambulatory knee arthroplasty.

Based on sample size calculations derived from prior literature, with an alpha error of 0.05 and a confidence interval of 95%, a minimum sample size of 92 patients was determined. To accommodate an anticipated refusal rate of 15–20%, the target number of included patients was set at 110 [15].

The Chinese-language questionnaires were distributed prior to any discussions concerning length of stay expectations or participation in preoperative knee arthroplasty education sessions. No information regarding ambulatory surgery or same-day discharge was disclosed before completing the questionnaire, and no promotional materials related to ambulatory knee arthroplasty were displayed in the medical office. Participants were instructed not to include their names on the questionnaire to minimize potential bias. Ethical approval for this study was obtained from the hospital research committee and the Institutional Review Board (IRB No. 202410004). Participation in the survey was entirely voluntary and not mandatory for any individual.

Demographic characteristics, including age, sex, occupation, region, and education level, were systematically gathered from the participants. Furthermore, comprehensive data regarding patients’ perceptions of ambulatory knee arthroplasty, such as familiarity with the procedure and beliefs about its potential to yield better outcomes (see Appendix A), were meticulously documented and analyzed. To ensure respondent anonymity, rigorous confidentiality measures were implemented throughout the survey process. Categorical variables were presented as frequencies accompanied by corresponding percentages. Univariate analyses were performed using Pearson’s chi-square tests (or Fisher’s exact tests when appropriate) to assess associations. Statistical significance was established at a threshold of *P* < 0.05. All statistical analyses were conducted using SPSS Statistics software (version 25; IBM Corp., Armonk, NY, USA).

## Results

A total of 195 patients consented to participate in the study. However, seven participants were unable to complete the survey successfully; two due to visual impairment and an inability to read the questionnaire, and five due to difficulties in using a smartphone. Consequently, a total of 188 participants completed the survey, yielding a high response rate of 96%. The demographic profile of the respondents indicated that 70% were female. The age distribution showed that 16% were younger than 60 years (*n* = 30), 58% were within the 60–70 years age group (*n* = 109), and 26.1% were above 70 years of age (*n* = 49). Eighty percent of the respondents (151 out of 188) reported no history of previous UKA or TKA. Of the patients who had previously undergone knee arthroplasty (*n* = 37), 65% had their surgery in an inpatient setting, while 35.1% had their procedure in an ambulatory setting. Notably, approximately 90% of these patients expressed satisfaction with their surgery outcomes and advocated for ambulatory surgery as a preferred option (Table 1).

**Table 1:** Survey participant characteristics (*n* = 188)

**Variable**		**Respondents, *n* (%)**
Age in years	< 60	30 (16)
	60–70	109 (58)
	> 70	49 (26.1)
Gender	Male	56 (29.8)
	Female	132 (70.2)
Occupation	Retire	51 (27.1)
	Worker	3 (1.6)
	Peasantry	117 (62.2)
	Freelance	5 (2.7)
	Others	12 (6.4)
Location setting	City	53 (28.2)
	County	31 (16.5)
	Town	19 (10.1)
	Village	85 (45.2)
Highest education level	Less than grade school	76 (40.4)
	Junior high school	63 (33.5)
	Senior high school	42 (22.3)
	College degree	7 (3.8)
	Graduate degree	0 (0.0)
Have you ever had a knee arthroplasty before?	Yes	37 (19.7)
	No	151 (80.3)
Surgery type	Inpatient surgery	24 (64.9)
	Ambulatory surgery	13 (35.1)
Are you satisfied with your last knee arthroplasty?	Very satisfied	17 (46)
	Satisfied	14 (37.8)
	Okay	5 (13.5)
	Dissatisfied	1 (2.7)
	Very dissatisfied	0 (0.0)
If you had to do it again, would you choose ambulatory knee arthroplasty?	Yes	33 (89.2)
	No	4 (10.8)

In total, sixty-eight percent of the patients (*n* = 128) demonstrated awareness of ambulatory surgery, as shown in Table 2. The awareness of ambulatory knee arthroplasty was found to be consistent across different demographic factors, as indicated by the lack of significant variation by age (X2 = 0.88, *P* = 0.64), sex (X2 = 1.76, *P* = 0.19), occupation (X2 = 3.93, *P* = 0.42), location (X2 = 2.13, *P* = 0.55), and education level (X2 = 0.96, *P* = 0.81). Among the 128 patients who were knowledgeable about ambulatory knee arthroplasty, sources of information varied. Specifically, 21.3% (*n* = 26) learned about it through prior surgical experiences, 53.1% (*n* = 68) from discussions with family and friends, 19.5% (*n* = 25) from orthopedic surgeons, and 7% (*n* = 9) from online platforms such as the internet or social media.

**Table 2:** Preferences regarding ambulatory knee arthroplasty

**Question**		**Respondents, *n* (%)**
Are you familiar with ambulatory knee arthroplasty?	Yes	128 (68.1)
	No	60 (31.9)
How have you heard about ambulatory knee arthroplasty?	Prior surgery	26 (20.3)
	Family/friends	68 (53.1)
	Doctor	25 (19.5)
	Internet/social media	9 (7)
Do you think ambulatory surgery is suitable for UKA or TKA?	UKA	44 (23.4)
	TKA	49 (26.1)
	Both	82 (43.6)
	Neither	13 (6.9)
Who do you think decide whether to have an ambulatory surgery?	Patients	15 (8)
	Doctor	136 (72.3)
	Anesthetist	3 (1.6)
	Health insurance department or insurance company	1 (0.5)
	Department	4 (2.1)
	Hospital	29 (15.4)
If the doctor gives you the option of ambulatory surgery, would you take it?	Very willing	97 (51.6)
	Maybe willing	52 (27.7)
	Unsure	27 (14.4)
	May not be willing	8 (4.3)
	Very reluctant	4 (2.1)
Do you think hospitals that can perform ambulatory surgery are better than those that do not?	Much better	66 (35.1)
	Better	81 (43.1)
	Equal	36 (19.2)
	Worse	3 (1.6)
	Much worse	2 (1.1)
Do you think surgeons who offer ambulatory surgery knee arthroplasty are better than those who do not?	Much better	70 (37.2)
	Better	82 (43.6)
	Equal	31 (16.5)
	Worse	4 (2.1)
	Much worse	1 (0.5)
Would you be willing to pay more out-of-pocket to have ambulatory knee surgery?	Very willing	12 (6.4)
	Maybe willing	51 (27.1)
	Unsure	81 (43.1)
	May not be willing	40 (21.3)
	Very reluctant	4 (2.1)
Would you be willing to travel further to have ambulatory knee surgery?	Very willing	13 (6.9)
	Maybe willing	48 (25.5)
	Unsure	60 (31.9)
	May not be willing	66 (35.1)
	Very reluctant	1 (0.5)
Would you be willing to wait longer to have ambulatory knee surgery?	Very willing	13 (6.9)
	Maybe willing	73 (38.8)
	Unsure	62 (33)
	May not be willing	39 (20.7)
	Very reluctant	1 (0.5)

**Fig. 1:**
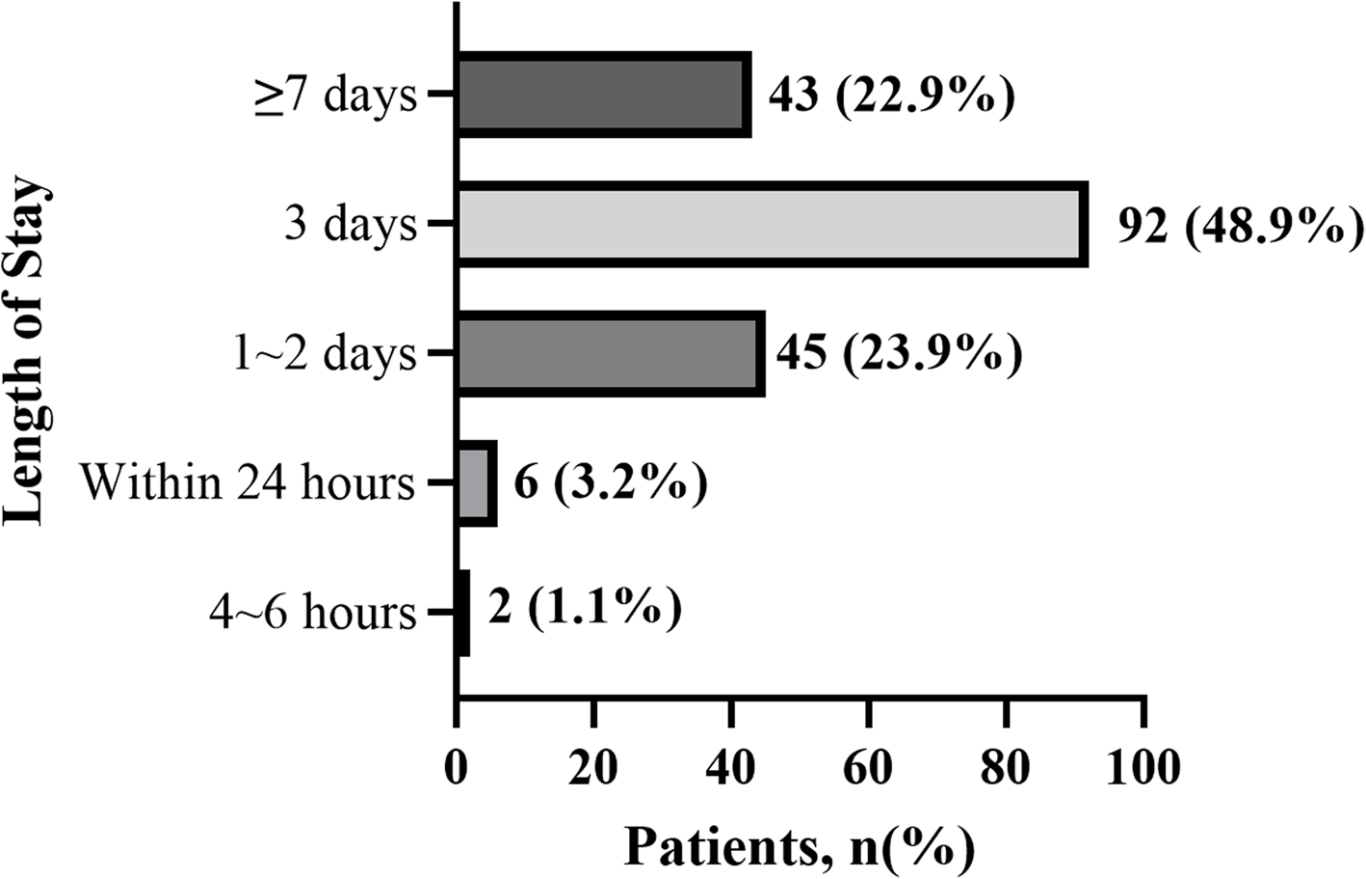
The length patients expected to stay in the hospital following knee arthroplasty

**Fig. 2:**
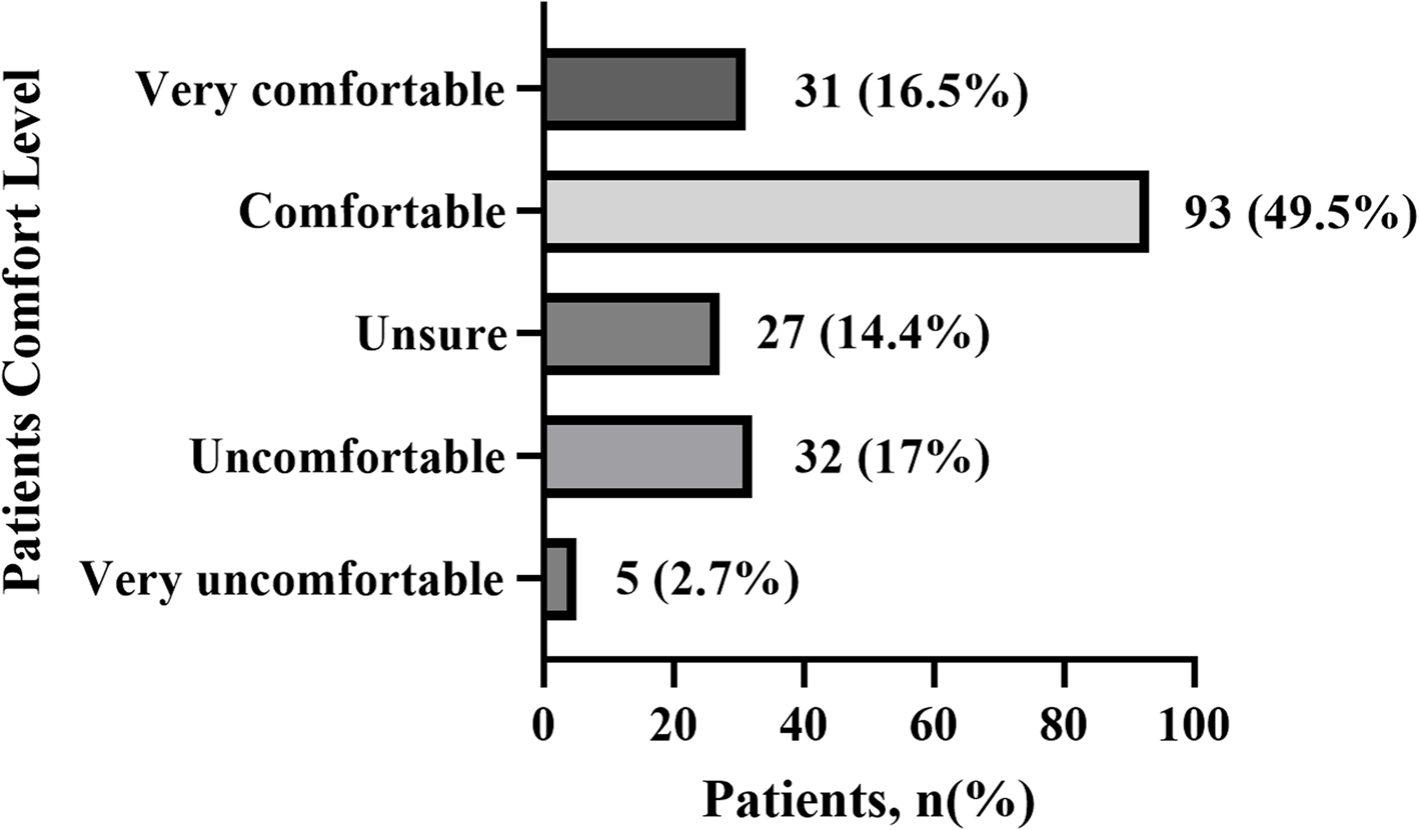
The comfort level of patients being discharged the same day or within 24 h after knee arthroplasty

According to Fig. 1, only 8 patients indicated an expectation of being discharged on the same day as their UKA or TKA surgery. Most respondents (48.9%) anticipated a discharge within 3 days, while approximately 22.9% expressed a preference for a hospital stay of 7 days or more. Of particular interest was the response to the question regarding comfort level with same-day or within 24 h discharge following knee arthroplasty, provided there is assistance available. Figure 2 illustrates that 66% of participants (*n* = 124) reported feeling either comfortable or very comfortable.

A significant proportion of patients, comprising 93% (*n* = 175), expressed the belief that ambulatory surgery was a suitable option for UKA or TKA. Among the respondents, the majority (72.3%) perceived the decision on whether to undergo ambulatory surgery as being primarily determined by the surgeon. Notably, if the surgeon presented the option of ambulatory surgery, approximately 79.3% of patients indicated a willingness to partake in it. However, it was observed that only 45% of patients were willing to endure longer waiting times, while a third of patients were open to paying more or traveling greater distances for ambulatory knee arthroplasty services.

According to Table 3, most patients indicated that they perceived ambulatory surgery centers to be as safe as or safer than inpatient units. Additionally, approximately 71.8% of the patients believed that opting for ambulatory surgery would result in improved outcomes characterized by reduced complications, infections, and pain, alongside a quicker recovery following UKA or TKA. Moreover, over 60% of the patients expressed the view that ambulatory surgery would not restrict their choice of knee prosthesis type.

**Table 3:** Expectations and perceived risks of ambulatory knee arthroplasty

Question	Respondents, *n* (%)				
	Definitely	Probably	Unsure	Probably not	Definitely not
Do you think that ambulatory knee arthroplasty would increase the cost?	14 (7.5)	42 (22.3)	71 (37.8)	45 (23.9)	16 (8.5)
Do you think that ambulatory surgery would lead to better results?	73 (38.8)	62 (33)	46 (24.5)	7 (3.7)	0 (0)
Do you think that ambulatory surgery would reduce the complications associated with knee arthroplasty?	40 (21.3)	59 (31.4)	77 (41)	11 (5.9)	1 (0.5)
Do you think that ambulatory surgery would reduce the pain after knee arthroplasty?	46 (24.5)	64 (34)	62 (33)	12 (6.4)	4 (2.1)
Do you think that ambulatory surgery would lead to faster recovery after knee arthroplasty?	52 (27.7)	67 (35.6)	57 (30.3)	9 (4.8)	3 (1.6)
Do you think that ambulatory surgery would limit the choice of knee prosthesis type?	18 (9.6)	52 (27.7)	80 (42.6)	28 (14.9)	10 (5.3)
Do you think that ambulatory surgery would reduce infections after knee arthroplasty?	29 (15.4)	63 (33.5)	74 (39.4)	18 (9.6)	4 (2.1)
Which location setting do you think is safer to have a UKA/TKA?	Ambulatory surgery center		Inpatient unit		Same
	50 (26.6)		81 (43.1)		57 (30.3)

**Fig. 3:**
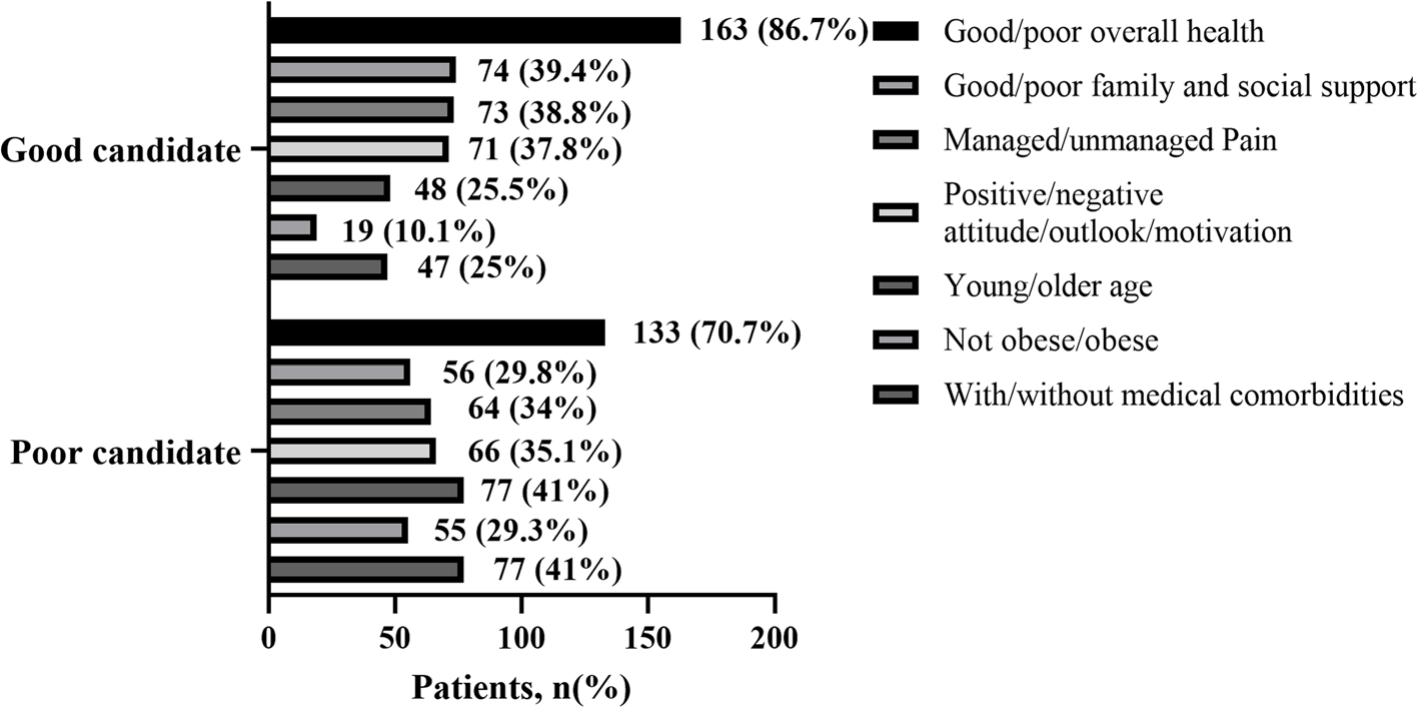
Patient perspectives on factors or characteristics making someone a good/poor candidate for ambulatory knee arthroplasty

In Fig. 3, responses to open-ended questions regarding the qualities that make an individual a suitable or unsuitable candidate for ambulatory knee arthroplasty were analyzed. Respondents consistently identified several key factors, including overall good health status, robust family and social support, effective pain management strategies, a positive attitude, presence of medical comorbidities, obesity status, and age, as critical considerations influencing candidacy for ambulatory knee arthroplasty.

## Discussion

The increasing prevalence of ambulatory knee arthroplasty in China can be attributed to the growing number of patients and the rising demand for medical services. Therefore, gaining a comprehensive understanding of patient perspectives regarding ambulatory surgery is of critical importance. In this study, we conducted an online survey using WenJuanXing to evaluate patients’ perceptions and preferences toward ambulatory knee arthroplasty. Our results indicated that over 68% of respondents were familiar with ambulatory surgery, with the majority (53.1%) obtaining information through family or peer networks. Awareness of ambulatory knee arthroplasty was consistent across various demographic factors, including age groups, gender, occupation, geographical location, and educational background. Although most Chinese patients (71.8%) initially preferred a longer hospital stay (≥ 3 days), they would likely consider ambulatory knee arthroplasty if provided with sufficient clinical evidence and robust social and familial support post-discharge, and would feel comfortable being discharged within 24 h.

A significant proportion of patients indicated their willingness to participate in ambulatory surgery, contingent upon the surgeon’s recommendation, and perceived hospitals or surgeons proficient in ambulatory surgery as being of higher quality. Furthermore, most patients believed that surgeries performed in an ambulatory surgery center were at least as safe, if not safer, than inpatient procedures. Additionally, over half of the patients considered ambulatory surgery to facilitate faster recovery and reduce complication rates, pain, and infection risks compared to inpatient surgeries. However, fewer than half of the patients were willing to tolerate extended waiting periods, and only a third were inclined to accept increased costs or travel longer distances for ambulatory surgery. These findings highlight the critical need to enhance the dissemination of information regarding ambulatory surgery. This can be achieved by improving patient-doctor communication through introducing ambulatory surgery during consultations, providing pre-operative educational materials and classes, and offering one-on-one counseling to address concerns. Moreover, promoting ambulatory surgery within hospitals via online platforms (websites and apps), hospital signage, information desks, and collaborating with insurance companies for favorable policies and joint promotion is essential. Finally, optimizing procedural processes, minimizing patient waiting times, and reducing economic burdens on patients are imperative to foster greater patient acceptance and satisfaction.

In March 2012, under the auspices of the Health Development Research Center of the National Health Commission, several domestic health administrative departments, research institutions, and leading medical facilities specializing in ambulatory surgery collaborated to establish the Chinese Association for Ambulatory Surgery (CASA). Subsequently, beginning in 2015, the Chinese government incorporated ambulatory surgery into the framework of national healthcare reform and introduced a series of policies. This strategic initiative played a pivotal role in promoting the standardization and development of ambulatory surgery practices across China [12]. Over the past year, numerous collaborative centers in China have actively explored the feasibility of implementing ambulatory joint arthroplasty. As an active member of CASA, our hospital has consistently performed over 10,000 knee arthroplasties annually. The advancement of ambulatory knee arthroplasty not only holds significant implications for enhancing hospital infrastructure but also contributes to optimizing medical insurance expenditures and improving overall societal welfare.

The success of ambulatory surgery depends on a multifaceted array of factors. In a position statement issued in 2018, the American Association of Hip and Knee Surgeons (AAHKS) and the American Academy of Orthopaedic Surgeons (AAOS) highlighted the appropriateness of conducting certain hip and knee arthroplasties in outpatient settings. They emphasized several critical elements essential for successful outpatient programs: 1) Rigorous patient selection; 2) Comprehensive patient education and effective management of expectations; 3) A robust social support network; 4) High proficiency and extensive experience of clinical and surgical teams; 5) An optimal facility environment conducive to enhancing surgical outcomes; 6) The implementation of evidence-based protocols for pain management, blood conservation, wound care, mobilization, and venous thromboembolism prophylaxis is essential [16]. It is universally acknowledged that meticulous patient selection serves as a fundamental determinant for the safe execution of ambulatory surgery. An ideal candidate generally exhibits characteristics such as younger age, good overall health, and access to a strong social support system [17]. According to findings from a nationwide register study involving 166,730 procedures conducted by Jensen et al. [18], approximately 48% of Danish hip and knee arthroplasty patients could potentially meet the criteria as day-case candidates. Furthermore, in carefully selected total joint arthroplasty (TJA) patients within academic settings, same-day discharge was considered feasible in nearly 98% of cases [19]. Even among unselected total hip arthroplasty (THA) and TKA patients, same-day discharge was achievable in about 15% of cases [20]. These data collectively underscore the feasibility of ambulatory knee arthroplasty for a significant proportion of patients.

However, the patient perspective regarding ambulatory joint arthroplasty remains underexplored. Adelani and Barrack [21] analyzed survey data from 346 TKA patients, revealing that only 4.9% of respondents believed they could have been discharged on the same day. When queried about the perceived benefits of ambulatory surgery, the most frequently cited advantages included reduced risk of infection (57.3%), improved sleep quality (46.9%), and a quieter recovery environment (42.7%). Similarly, Meneghini et al. [13] surveyed 110 patients who underwent TJA, assessing their awareness and perceptions of ambulatory arthroplasty. Most participants considered ambulatory surgery centers to be as safe as hospitals for surgical procedures and anticipated a postoperative stay of 1 to 2 days. In China, where the practice of ambulatory knee arthroplasty is gaining traction, understanding patient perspectives is equally essential. However, there has been limited research into Chinese patients’ views on this procedure. In our study, we evaluated survey data from 188 patients undergoing UKA or TKA. Despite the relatively low educational attainment of Chinese patients, 68% demonstrated awareness of ambulatory knee arthroplasty, and nearly 80% expressed willingness to undergo such a procedure if recommended by their surgeons. Furthermore, over 70% of patients believed that ambulatory surgery would result in enhanced outcomes, including lower complication rates, reduced pain, and decreased infection risks. Based on these findings, we propose that implementing ambulatory knee arthroplasty in China is both feasible and promising.

Social support is a critical determinant of success in ambulatory surgery. Our study demonstrated that more than 70% of patients preferred to stay in the hospital for three days or longer, with only eight patients expressing a willingness to be discharged within 24 h post-surgery. Notably, 66% of patients indicated that they would feel comfortable being discharged on the same day or within 24 h if sufficient post-discharge care were provided. Consequently, it is essential to evaluate a patient’s living circumstances and social support network prior to surgery, with an emphasis on identifying individuals capable of directly assisting in the patient’s recovery process. The surgical team must proactively engage and educate both the patient and their social support network. Despite variations in home environments and cultural backgrounds, patients’ attitudes toward ambulatory surgery appear to be consistent. It is imperative to recognize that patients lacking robust social support are not optimal candidates for ambulatory surgery [17].

Our study demonstrated several key strengths. Achieving a response rate of 96%, which closely aligns with the 98% response rate reported in previous literature [13], indicated robust engagement from participants. Furthermore, the respondents consisted entirely of patients scheduled for knee arthroplasty, enhancing the representativeness of this group’s perspectives on related disease treatment. However, several limitations warrant acknowledgment. Firstly, this study exclusively surveyed patients at a tertiary orthopaedic hospital, potentially limiting the generalizability of findings to the broader Chinese population. Nonetheless, the study served as an initial exploration into patient attitudes towards ambulatory surgery, particularly as ambulatory knee arthroplasty gains traction in China. Secondly, while the study boasted a high response rate, the sample size was relatively small, comprising only 195 patients. Despite this, the sample size remains sufficient to provide insights into the average patient’s attitudes towards ambulatory surgery. Lastly, the use of mobile devices for questionnaire administration might introduce respondent bias due to potential selectivity in the completion process. However, as the sample size grows, any such bias is expected to diminish over time.

## Conclusions

Our study findings reveal that most Chinese patients initially prefer a longer hospital stay (≥ 3 days); however, with adequate clinical evidence and strong social and familial support post-discharge, most patients would be willing to consider ambulatory knee arthroplasty and comfortable with a discharge within 24 h. Despite this willingness, a significant proportion of patients (one-third) currently lack awareness regarding ambulatory knee arthroplasty, highlighting the need for enhanced patient education and awareness campaigns moving forward. As the prevalence of ambulatory procedures rises in China, it is imperative to prioritize increasing patient understanding of the ambulatory surgery process, along with its associated benefits and risks, to ensure optimal patient outcomes and experiences in ambulatory care settings.

## Supplementary Information

Additional file 1

## Data Availability

The datasets used or analyzed during the current study are available from the corresponding author on reasonable request.

## Abbreviations

UKA: Unicompartmental knee arthroplasty
TKA: Total knee arthroplasty
CASA: China Ambulatory Surgery Alliance
AAHKS: American Association of Hip and Knee Surgeons
AAOS: American Academy of Orthopaedic Surgeons
TJA: Total joint arthroplasty
