# Supplementary material for: Patient perceptions regarding ambulatory knee arthroplasties in China
Source: Arthroplasty. 2025 Jun 9;7:28. doi: 10.1186/s42836-025-00316-z (PMC12147369; doi:10.1186/s42836-025-00316-z)
Supplement: Supplementary file 1 — Additional file 1 [file 42836_2025_316_MOESM1_ESM.docx]

**Appendix A**

*Patient Perspectives on Ambulatory Knee Arthroplasties （English Version）*

1. Gender: a) Male b) Female

2. Age: Years old

3. Occupation:

a) Retire b) Worker c) Peasantry d) Freelance e) Others

4. Location setting:

a) City b) County c) Town d) Village

5. Highest education level:

a) Less than grade school b) Junior high school c) Senior high school d) College degree e) Graduate degree

6. Have you ever had a knee arthroplasty before?

a) Yes (If yes, In-patient or Ambulatory surgery) b) No (*Turn to question 9*)

7. Are you satisfaction with your last knee arthroplasty?

a) Very satisfied b) Satisfied c) Okay d) Dissatisfied

e) Very dissatisfied

8. If you had to do it again, would you choose ambulatory knee arthroplasty?

a) Yes b) No

9. Are you familiar with ambulatory knee arthroplasty?

a) Yes b) No (*Turn to question 11*)

10. How have you heard about ambulatory knee arthroplasty?

a) Prior surgery b) Family/friends c) Doctor d) Internet/social media e) Television f) Others, please list:

11. Do you think ambulatory surgery is suitable for UKA or TKA?

a) UKA b) TKA c) Both d) Neither

12. Who do you think decide whether to have an ambulatory surgery?

a) Patients b) Doctor c) Anesthetist d) Health insurance department or insurance company e) Department f) Hospital

13. If the doctor gives you the option of ambulatory surgery, would you take it?

a) Very willing b) May be willing c) Unsure d) May not be willing

e) Very reluctant

14. Do you think that ambulatory surgery would lead to better results?

a) Definitely b) Probably c) Unsure d) Probably not

e) Definitely not

15. Do you think that ambulatory surgery would reduce the complications associated with knee arthroplasty?

a) Definitely b) Probably c) Unsure d) Probably not

e) Definitely not

16. Do you think that ambulatory surgery would reduce the pain after knee arthroplasty?

a) Definitely b) Probably c) Unsure d) Probably not

e) Definitely not

17. Do you think that ambulatory surgery would lead to faster recovery after knee arthroplasty?

a) Definitely b) Probably c) Unsure d) Probably not

e) Definitely not

18. Do you think that ambulatory surgery would limit the choice of knee prosthesis type?

a) Definitely b) Probably c) Unsure d) Probably not

e) Definitely not

19. Do you think that ambulatory surgery would reduce infections after knee arthroplasty?

a) Definitely b) Probably c) Unsure d) Probably not

e) Definitely not

20. Which location setting do you think is safer to have an UKA/TKA?

a) Ambulatory surgery center b) Inpatient unit c) Same

21. Do you think hospitals that can perform ambulatory surgery are better than those that do not?

a) Much better b) Better c) Equal d) Worse e) Much worse

22. Do you think surgeons who offer ambulatory surgery knee arthroplasty are better than those that do not?

a) Much better b) Better c) Equal d) Worse e) Much worse

23. In your opinion, which factors or characteristics make someone a good candidate for ambulatory knee arthroplasty? (multiple-choice)

a) Good overall health b) Good family and social support c) Managed pain d) Positive attitude/outlook/motivation e) Younger age f) Not obese g) Without medical comorbidities

24. In your opinion, which factors or characteristics make someone a poor candidate for ambulatory knee arthroplasty? (multiple-choice)

a) Poor overall health b) Poor family and social support c) Unmanaged pain d) Negative attitude/outlook/motivation e) Older age

f) Obese g) With medical comorbidities

25. How long would you expect to stay in the hospital following knee arthroplasty?

a) 4~6 hours b) Within 24 hours c) 1~2 days d) 3 days

e) ≥7 days

26. Assuming you have someone to assist you, how comfortable would you be being discharged the same day or within 24 hours after knee arthroplasty?

a) Very comfortable b) Comfortable c) Unsure d) Uncomfortable

e) Very Uncomfortable

27. Would you be willing to pay more out-of-pocket to have ambulatory knee surgery?

a) Very willing b) May be willing c) Unsure d) May not be willing

e) Very reluctant

28. Would you be willing to travel further to have ambulatory knee surgery?

a) Very willing b) May be willing c) Unsure d) May not be willing

e) Very reluctant

29. Would you be willing to wait longer to have ambulatory knee surgery?

a) Very willing b) May be willing c) Unsure d) May not be willing

e) Very reluctant

30. Please feel free to add any additional comments you may have here:

**You are finished with the survey. Thank you for your time. All the best to you.**
